# Cognitive Keys in Psychophysical Estimation of Chemosensory Perception in University Students

**DOI:** 10.3390/foods10123134

**Published:** 2021-12-17

**Authors:** Laura María Martínez-Sánchez, Cecilio Parra-Martínez, Tomás Eugenio Martínez-García, Concha Martínez-García

**Affiliations:** 1 Department of Didactics of Physical, Plastic and Musical Education, Faculty of Education Sciences, University of Cádiz, 11519 Puerto Real, Spain; 2 Department of Chemistry, Faculty of Experimental Sciences, University of Huelva, 21007 Huelva, Spain; cecilio.parra@dqcm.uhu.es; 3 Department of Internal Medicine, Juan Ramón Jiménez University Hospital, 21005 Huelva, Spain; teugenio.martinez.sspa@juntadeandalucia.es; 4 Department of Social, Developmental and Educational Psychology, Faculty of Education, Psychology and Sports Sciences, University of Huelva, 21007 Huelva, Spain

**Keywords:** taste perception, psychophysical measures, absolute threshold, astringency

## Abstract

Psychophysical methods allow us to measure the relationship between stimuli and sensory perception. Of these, Detection Threshold (DT) allows us to know the minimum concentration to produce taste identification. Given this, we wonder whether, for example, wine tasting experts are more capable of perceiving their sensory properties than other people, or whether they can distinguish them because they are better able to “describe” them. To verify this, this study analyses the influence of having prior knowledge of the name astringency and, failing that, to detect it and distinguish it between the four basic tastes. One-hundred-and-sixty-two university students with an average age of 19.43 (SD = 2.55) years were assigned to three experimental conditions: an experimental group (G.2) without previous knowledge of the name astringency and with alimentary satiety, and two control groups, both with previous knowledge of the name, these being G.1, with satiety, and G.3, with hunger. DT was collected for the four basic tastes and astringencies. Results showed significant differences in the identification of astringency, being the least identified experimental group with respect to the control groups. It is striking that G.2, without prior knowledge of the name, identified astringency as a bitter taste in most cases. This supports our hypothesis of the importance of attending to linguistic cognitive processes when psychophysically estimating taste in humans.

## 1. Introduction

Advancing the understanding of psychological processes that are involved in the perception of food by consumers, for their preference of choice or rejection, has been seeing an increasing trend in recent years; however, there are numerous factors that influence the sensory mechanism, and there are also difficulties in its analysis methodology, so we are still far from elucidating the guidelines that would give concordance between consumer expectations and their sensory experience of what is consumed [1]. In relation to studies that focus on the ingredients that promote an unwanted taste sensation, mostly in order to minimize or balance them, we found studies that delve into the mechanisms involved in the perception of astringency, usually described by experimental subjects as a sensation of dryness and roughness in the mouth [2].

Essentially, the tactile and haptic sensation of astringency is due to the ability of some phenolics, such as tannins, to bind to salivary proteins, forming macromolecular complexes that precipitate, causing the loss of their lubricating capacity, and thus contributing to a dry mouth sensation and roughness [3,4,5]. Among the best-known astringent foods would be cheese, tea, walnut skins, chocolate and numerous fruits, but in wine, astringency has great relevance because its balanced level could distinguish a great wine from a bad one, given that astringency enhances the flavors of red wines and prolongs their finish [6,7,8].

On the sensory perception of taste, previous literature has shown that it is a trans modal interactive process that is influenced not only by the stimulation of the external environment, type of taste stimuli, age, sex, and other factors [9], but also by different internal brain states and different memory loads [10]. However, questions remain about how the brain processes and modulates the perception of taste.

In this sense, this processing is even more unknown when the perception of taste is related in a linguistic key (conscious) for its interpretation, labelling, and storage in our cognitive repertoire, being essential for its recognition in future detection and for taste research [7,11], and this complication increases when the homeostatic state of the subject is attended to at the time of the study.

The aim of the present study was to address some aspects involved in taste identification. The first objective was to determine Detection Thresholds (DT) for the four basic tastes of sweet, salty, bitter, and acidic in university students. The second objective was to compare DT in conditions of hunger and food satiety, and the third objective was to analyze the possible influence of prior knowledge of the name astringency for its recognition, as opposed to not having it. The study was proposed with young subjects due to optimal sensitivity of their taste buds at this stage of development. Participants were distributed in three groups with the experimental conditions of food hunger/satiety and knowledge/ignorance of the word astringency.

## 2. Materials and Methods

### 2.1. Participants

The participants, 17–32 years old with an average age of 19.43 (SD = 2.55) years, consisted of 162 students (75.3% women) from the University of Huelva (Spain) that were distributed into 3 groups: the satiety control group was Group 1 (G.1) (*n* = 54) with 13 males and 41 females, the experimental group was Group 2 (G.2) (*n* = 53) with 15 males and 38 females, and hunger control group was Group 3 (G.3) (*n* = 55) with 12 males and 43 females. The inclusion criteria were being over 17 years of age and providing written informed consent by students established by the Bioethics Committee of the University of Huelva, following the guidelines of the Declaration of Helsinki. The exclusion criteria were having an impairment of taste or olfactory abilities and undertaking treatment with drugs or psychotropic drugs that could alter these capacities.

### 2.2. Materials

Recommendations for flavor stimuli detection by the International Organization of Vine and Wine (OIV) [12] were followed to select solution concentrations (stock solutions of sucrose 10 g/L, tartaric acid 0.5 g/L, sodium chloride 2 g/L, and quinine sulphate 5 mg/L). In order for solutions to be identified by all study participants, the concentrations were increased to 50%, while to find the lowest thresholds (DT) that these young participants were able to perceive, concentrations were reduced down to 2 g/L sucrose, 0.1 g/L of tartaric acid, 1 g/L of sodium chloride, and 2 mg/L of quinine sulphate. The fifth stock solution was 1 g/L of tannic acid for the detection of astringency (Table 1).

A concentrated solution for each flavor tested was prepared following the guidelines of Norma ISO 3972 [13]. These solutions were: 20 g/L of sucrose for sweet taste, 1 g/L of tartaric acid for acidic taste, 4 g/L of sodium chloride for salty taste, and 10 mg/L of quinine sulphate for bitter taste. Solutions with decreasing concentrations were prepared from these solutions for each taste.

A detection record sheet prepared for this study was used, in which the perceived flavors were recorded, noting, next to the dissolution number (from tasting number 1 to tasting number 5 in each exercise), the name of the flavor they thought they perceived.

### 2.3. Procedure

The present work was a quasi-experimental study, cross-sectional, and was carried out with intentional sampling. Groups designs were non-equivalent and with reverse treatment. This means that in one group (G.1), a treatment is introduced that is expected to produce an effect in the positive direction (from the hypothesis raised), and in the other group (G.2), the opposite treatment is introduced, or conceptually opposite, which is expected to reverse the effect pattern [14,15]. In this study, the “treatment” was to explain the meaning of astringency sensation in G.1, hoping that this would provide a better identification of said sensation. The last group (G.3) was the control group for the satiety/hunger variable. The experimental conditions of the groups in this study were: G.1, without food restriction and with advance information of the name “astringency”. This information about the meaning of word astringency was given during the explanation prior to the tasting. The characteristics of G.2 were without food restriction and without anticipation of that name, because this information was not given during the explanation prior to the tasting. The last group, G.3, with food restriction and with anticipation of the name “astringency”, received the same information as G.1.

All groups carried out identical tasting exercises, A, B, and C, each one with five glass cups to test, where some flavor was repeated but with different concentrations in exercises A and C, whereas in B, the sensation of astringency was introduced along with the four basic flavors (Table 1). On a sheet of paper, the corresponding numbers of each solution in each exercise were written and placed just below each corresponding glass cup. When exercise A was finished, solutions were removed and new solutions for exercise B were served. The same procedure was carried out for exercise C. The order in which the cups were tested by participants is detailed in Table 1. Participants had 1 L of water available per row (three participants per row) and a paper cup for washing out their mouths between tastings. Before starting each exercise, they were asked to repeat mouth washes until the palate was cleaned. They were also instructed to rinse their mouths after each tasting.

The instructions given to participants about their nutritional status before the experiment are summarized below: the two satiety groups had to eat abundantly before going to the classroom-laboratory, highlighting their importance for the theoretical–practical tasting, their schedules coinciding after their usual breakfast time (G.1) and lunch (G.2). The food content was freely chosen by participants (because they knew about their allergies, habits, and food preferences) to facilitate a greater satiety state. When they arrived at the classroom-laboratory, all the materials had already been arranged in their places and the tasting began after explanation. For the hunger group (G.3), the instructions were different: they had to go to the classroom-laboratory just at the end of their previous 2-h class, at 11:00 a.m., which implied cognitive wear (energy consumption: glucose), and they should not take any food except water, also highlighting its importance. Furthermore, once in the classroom, the start of the distribution of solutions was delayed for a further 1 h, thus ensuring a fast of more than 3 h.

The summary of the instructions given is the following:

- G.1 (9:30 a.m. *): go to the classroom-laboratory at 9:00 a.m., after breakfast.
  Upon arriving at the place, participants had already distributed all the necessary material (informed consent, information sheets detection, paper mats to be numbered, glassware, bottles of water for mouth rinsing between tastings, dump bins, etc.) and, in the first half hour, a brief theoretical explanation of the lingual areas with the highest concentration of chemical receptors for each basic flavor was offered.
  During the experiment in exercise B, it was reported in this group that astringency was included in their tasting along with the four basic flavors.
- G.2 (4:00 p.m. *): go to the classroom-laboratory at 3:30 p.m., after lunch.
  Upon arriving at the place, participants had already distributed all the necessary material, and in the first half hour, they were offered an identical theoretical explanation as that given to G.1.
  During the experiment in exercise B, it was reported in this group that, along with the basic flavors, a “surprise” taste was included to identify among the five solutions.
- G.3 (12:00 p.m. *): do not consume any food or drink, except water, from 9:00 a.m. and go to the classroom-laboratory at 11:00 a.m.
  In the hour prior to the experiment, from 11 a.m. to 12 p.m., the necessary material was distributed, and they were offered an identical theoretical explanation as that given to G.1.
  During the experiment in exercise B, it was reported in this group that astringency was included for their tasting along with the four basic flavors.
  (*): Start time of the experiment.

In exercise A, the highest concentration dissolution was presented; in exercise B, different descending concentrations were available and included the solution of 1 g/L of tannic acid for the perception of astringency; and finally, in exercise C, the lower concentrations of flavors were presented.

Within each exercise, the order of administration of the flavors was randomized, which were then numbered from 1 to 15 to be distributed identically in the three groups (Table 1). All solutions were supplied in 30 mL/cup.

### 2.4. Statistical Analyses

All statistical tests were performed using SPSS, vers. 20.0 (IBM Corp., Armonk, NY, USA), Statistics for MAC [16]. Significance was set at *p* < 0.05. For the description of qualitative variables such as sex and DT, absolute frequencies (n) and relative frequencies (%) were calculated, and for the age variable, the average was calculated and the Kolmogorov–Smirnov test was used to assess the normality of the sample. For the age variable, the hypothesis of equality between means was assessed with one-way ANOVA when samples followed normal distribution; otherwise, the Kruskal–Wallis test was applied. For the qualitative variables, Pearson’s Chi-square test (χ^2^) was used. In the analysis of the significance between the qualitative variables relating to astringency and four basic tastes, contingency coefficient was used.

## 3. Results and Discussion

### 3.1. Detection Threshold (DT) for the Four Basic Tastes

The participants’ average age was 19.43 years (SD = 2.55) and there were no significant differences in age among groups (*p* = 0.07). Although there was a higher percentage of women (75.3%), they were well distributed between groups, and sex had no influence on the groups (χ^2^ = 0.627; *p* = 0.731).

Detection threshold results for the four basic tastes are shown in Table 2.

Absolute threshold (AT) results for basic tastes are presented in Table 2. For the sweet flavor, the lowest concentrations detected by participants were 2 g/L for 63 of them (38.9%), 5 g/L for 90 students (55.6%), and the rest showed an AT of 10 g/L. It should be noted that all participants detected a sweet taste with the highest concentration (20 g/L of sucrose), but for none of them was this concentration their lowest threshold, their AT being any of the other three low values (2, 5 or 10 g/L), as mentioned above. The salty flavor was detected at a minimum concentration (1 g/L of NaCl) by 91.4% of participants. The bitter flavor had a decreasing AT range from 2 to 10 mg/L of quinine sulphate and was not detected by 6.8% of participants. For half of the participants (50.6%), AT for acidic was 0.5 g/L of tartaric acid and for 46.3% of them they were concentrations of 0.1 g/L and 0.75 g/L.

In this first objective, note that in addition to the fact that all participants detected the highest concentration for the sweet taste, 94.5% of them identified the flavor in the two lowest concentrations, showing good sensitization for sweetness. Preference for sweet tastes in humans is innate and universal [17,18]. While with potential food it has to be learnt what is nutritious, safe or toxic, a sweet taste in human beings and other species is the only element of food that seems to be an innate preference [19,20]. In the case of a salty taste, where DT was the lowest concentration for most of the participants, results showed that it is a well-identified flavor with the minimum concentration. Salty is an innately accepted flavor [21], but in a moderate level, given that a high concentration is rejected [20]. Our DT results for acidic and bitter indicated that the flavors are detected in medium and lower concentrations, which is reasonable considering that bitter and acidic tastes are associated with food aversion [21].

Detection thresholds tend to increase with age as a result of multiple factors related to age [22], and this means elderly people need a greater concentration of tastes to identify them. In a study carried out by Mojet et al. [23], taste thresholds were compared between elderly and young people. Our DT results for sweet tastes are comparable with the mean thresholds obtained by Mojet in young participants. In the case of salty tastes, young participants of Mojet’s study obtained lower mean thresholds as the range for taste detection was broader. In our case, the vast majority of the participants obtained a DT for salty taste of 1 g/L, but surely, they are capable of showing lower thresholds with lower concentrations. However, Jeon’s study [24] showed similar results to us for salty DT in young participants. The study of Louro et al. [20], where taste thresholds were evaluated, is not comparable with our results because their concentration range was higher for sweet and salty, and the average age of participants was also higher than ours. Substances used for evaluating acidic and bitter thresholds in the mentioned studies were not the same as ours and were not comparable. Variables such as genetics and variation in saliva composition, in addition to the lack of a consensual protocol to determine flavor thresholds make it difficult to compare the results of different studies.

### 3.2. Detection Thresholds (DT) in Conditions of Hunger and Food Satiety

**Figure 1:**
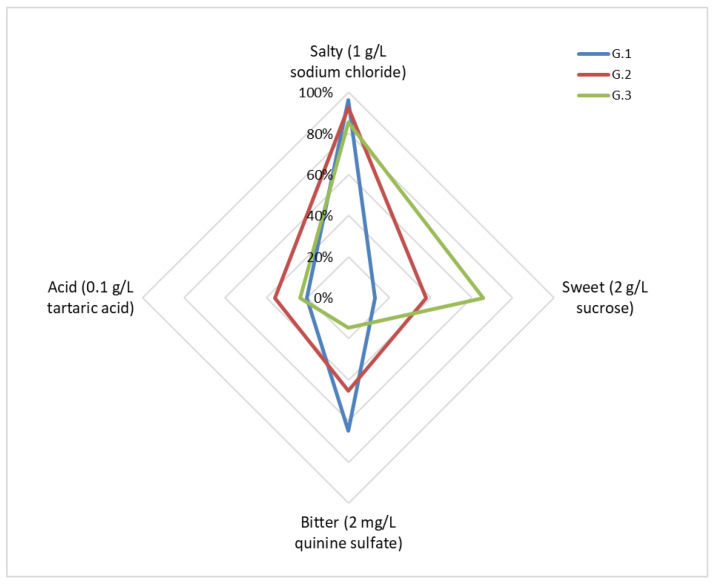
Percentage of detection for the lowest concentration of basic tastes in each group.

The corresponding analysis of different experimental conditions of groups (Table 2 and Figure 1) showed significant differences regarding the AT of sweet (χ^2^ = 33.293; *p* < 0.01) and bitter (χ^2^ = 32.429; *p* < 0.01) flavors where G.3, the hunger control group, had the lowest AT for sweet (2 g/L of sucrose) and where G.3 had the highest AT for bitter (5 mg/L of quinine sulphate) tastes. There were no significant differences between groups for salty (χ^2^ = 9.148; *p* = 0.111) or acidic (χ^2^ = 10.558; *p* = 0.216) flavors and ATs were 1 g/L of NaCl and 0.5 g/L of tartaric acid, respectively, as we mentioned before.

The only food-restricted group, G.3, was the one that could better detect sweet tastes at the minimum concentration. Group 3 was responding to an energy need by a homeostasis mechanism that increases the search for that flavor [25]. By contrary, in a state of food satiety, a process of ceasing food intake is activated, where one of the main factors is that food stops being pleasant [26].

Moreover, the same food-restricted state showed the highest DT for bitter tastes, and following the strategy for homeostasis control, this would allow increasing the intake of energy substances. Groups with food satiety presented the reverse strategy (Figure 1): low DT for bitter tastes in order to restrict dietary intake [25].

Hanci and Altun [27] reported significant differences between sensitivity to sweet, salty, and acidic tastes in subjects with different nutritional states, showing higher sensitivity in hunger than in satiety, as they expected. However, the sensitivity to bitter tastes was significantly higher in satiety. These results support our data for sweet and bitter, but do not coincide for salty and acidic tastes. Even so, some authors pointed out that taste sensitivity could be related to the perception of nutrients depending on the nutritional state (satiety or hunger) [28,29,30].

In the present study, results for salty and acidic tastes showed not to be influenced by restriction or satiety conditions. The appetite for salt is directly related to the lack of sodium (Na^+^), a concept of specific hunger, and cannot be satisfied with the ingestion of other cations, such as Ca^2+^ or K^+^. For acid, the degree of acidity corresponds to the pH concentration within taste cells where the H^+^ ligand blocks the selective K^+^ channel to depolarize the neuron [31]. For this reason, we believe that fasting would be necessary prolonged over time (more than 3 h) to cause any effect in salty and acidic thresholds. Participants were healthy young subjects whose levels of cellular Na^+^ and H^+^ ions do not seem to have been affected, thus explaining that there were no significant differences between two nutritional conditions.

To verify these results on salty and acidic thresholds, future studies would be necessary; for example, a study in which a control group similar to the one studied here will be compared with a clinical group (for example, patients with anorexia nervosa), whose mean ages were similar. Variations in their DT could be verified with biochemical analysis in both groups according to the conditions of greater or lesser food restriction.

### 3.3. Astringency Detection and Its Prior Knowledge

**Figure 2:**
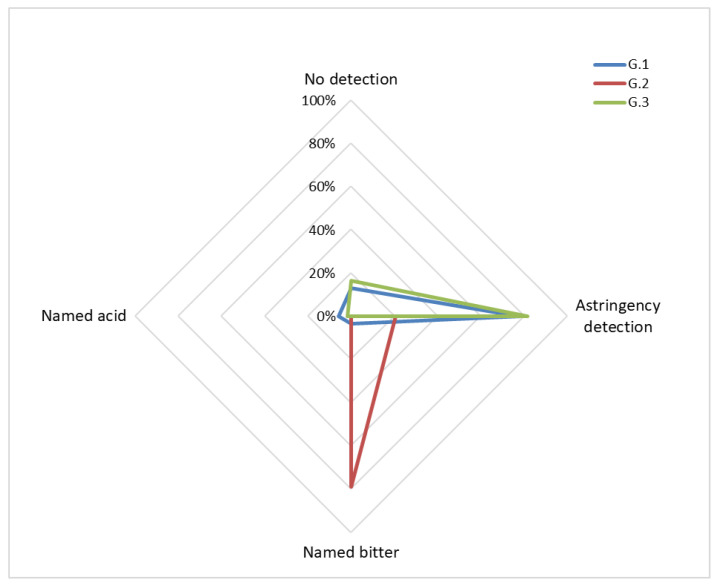
Percentages of astringency identification by groups.

Identification of astringency was clearly different between groups (χ^2^ = 111.037; *p* < 0.01) and was higher in subjects who knew the name of this sensation compared with subjects in whom the word astringency was unknown (77.8% and 81.8% vs. 20.8%) (Table 3 and Figure 2). These results were independent of the satiety or hunger state of the participants (χ^2^ = 3.345; *p* = 0.377) (Figure 2). There were a higher association between astringency detection and the knowledge of the word astringency (contingency coefficient = 0.638). Of all participants who were correct in astringency detection, 88.9% belonged to the groups that knew the word astringency. Curiously, the astringent substance was identified as a bitter taste in those participants without knowledge of this word (79.2%).

Taste is a cognitive construct, supporting the existence of a functional flavor system characterized by a dependence associated with learning [32]. Our study population corresponds to a young population, and although they have had learning of the tactile and haptic sensation of astringency, due to the common and varied foods that produce it, in most cases at that age they do not know the term “astringency” to identify it. If we had asked them to describe it, they would surely say that they found it rough and felt a dry mouth [2], but they have had difficulty naming it as “astringency”, perhaps because they are not experts, nor are they regular consumers of red wine, which is the context in which this term is most used at the popular level.

In fact, in our experimental group, as the subjects did not know the word astringency, they identified this sensation with a taste for which they did have a name in their cognitive repertoire—bitter—and although both can cause sensations associated with food aversion, it is well established that each one has its own characteristic sensory properties [2].

These considerations raise a reflection that these cognitive keys should not be neglected in experimental subjects when their chemosensory perception is being measured, as errors could be made in the answer register, where they will appear that they have not been detected due to the non-emission of response, when in fact they did detect it but did not know how to name it.

Our results support what was described in Croijmans et al. [33] regarding the fact that a professional practical training in wine was necessary to improve the images of the students as a wine tester. This specific training affected the ability to imagine the color, smell, and taste of wine, but not of everyday objects. In our study, we started with a group that had experience—experts—in the four basic flavors, because of everyday foods that we all consume, and also on the astringent sensation due to the consumption of other habitual flavors, such as vinegar in salad, persimmon, lemon, dark chocolate, etc. As in the study by Croijmans et al., specific training on the name of the basic tastes (sweet, bitter, salty, and acidic) is given in homes during the development and thus they are learned at the same time that we learn to eat and ask for our food preferences. This is not the case for astringency, for example, as when we bite into an unripe fruit during this daily learning; it is more common to name our sensation as “strong or rough”, instead of calling it “astringent”. Our results show that when the participants were given specific practical training (G.1 and G.3), facilitating the name of astringency and offering them practical examples of the foods that cause it (remembering, or imagining, its sensation in the mouth), they can recognize it by calling it astringency and distinguish it from bitter and acidic tastes. On the contrary, when such information is not offered to them (G.2), they are not able to identify it, despite the previous experience of that sensation by the foods that cause it.

In the aforementioned study, students becoming experts in wine (novices) had an average age (43.6 years) greater than the participants of the present study and also conducted a first phase with wine professionals whose average age was 48.7 years. In both cases, the experience with specific training was necessary for the best description of the evaluated aspects of the wine with respect to their control participants, but they did not differentiate between them for other aspects not related to the wine. For this reason, it is more important that our participants did not know the word astringency since, due to their age, they were not habitual wine consumers, which is the context where it is most named and were specific training was necessary since, without it, they erred in its identification in the tasting.

## 4. Conclusions

Results for the absolute threshold (AT) in the young population studied showed that both sweet and salty tastes were detected at the lowest concentrations tested and bitter and acidic tastes were detected at low and medium concentrations.

The nutritional status of participants conditioned the AT for sweet and bitter tastes, but not for salty and acidic tastes. In a hungry state, there was a lower threshold for sweet but a higher threshold for bitter tastes, whereas in a food satiety state, these thresholds were higher for sweet and lower for bitter tastes.

Results showed significant differences in the identification of astringency, with the experimental group (G.2) being without prior knowledge of the word astringency, identifying it the least, with respect to the control groups (G.1 and G.3) that did know the word. This supports our hypothesis of the importance of attending to cognitive processes linguistically when psychophysically estimating taste in humans.

We conclude that nutritional status and theoretical concepts related to flavors can influence the process of taste perception. This should be considered in psychophysical studies on the chemical sense perception to reduce false negatives (incorrect detection), particularly when flavors are erroneously named but physiologically correctly processed.

## Figures and Tables

**Table 1:** Composition and descending order of solutions ^(1)^ of four basic tastes and astringency in three exercises of theoretical–practical tasting.

Exercise A	Exercise B	Exercise C
(1). 2 g/L sodium chloride	(6). 0.75 g/L tartaric acid	(11). 0.1 g/L tartaric acid
(2). 1 g/L tartaric acid (acidic)	(7). 10 g/L sucrose	(12). 2 g/L sucrose
(3). 10 mg/L quinine sulphate (bitter)	(8). 5 mg/L quinine sulphate	(13). 5 g/L sucrose
(4). 20 g/L sucrose (sweet)	(9). 1 g/L sodium chloride	(14). 0.5 g/L tartaric acid
(5). 4 g/L sodium chloride (salty)	(10). 1 g/L tannic acid (astringent)	(15). 2 mg/L quinine sulphate

^(1)^ Numbers with parentheses: order of administration. Underlined: the biggest concentrate.

**Table 2:** Summary DT results by group for basic tastes.

			Student Group			
Taste	Solution		Group 1	Group 2	Group 3	Total
Sweet threshold	2 g/L sucrose	Head count	7	20	36	63
		Within-group comparison (%) ^1^	13.0	37.7	65.5	38.9
		Between-group comparison (%) ^2^	11.1	31.7	57.1	100.0
	5 g/L sucrose	Head count	41	31	18	90
		Within-group comparison (%) ^1^	75.9	58.5	32.7	55.6
		Between-group comparison (%) ^2^	45.6	34.4	20.0	100.0
	10 g/L sucrose	Head count	6	2	1	9
		Within-group comparison (%) ^1^	11.1	3.8	1.8	5.6
		Between-group comparison (%) ^2^	66.7	22.2	11.1	100.0
	20 g/L sucrose ^3^	Head count	-	-	-	162
Salty threshold	1 g/L NaCl	Head count	52	49	47	148
		Within-group comparison (%) ^1^	96.3	92.5	85.5	91.4
		Between-group comparison (%) ^2^	35.1	33.1	31.8	100.0
	2 g/L NaCl	Head count	1	3	4	8
		Within-group comparison (%) ^1^	1.9	5.7	7.3	4.9
		Between-group comparison (%) ^2^	12.5	37.5	50.0	100.0
	4 g/L NaCl	Head count	1	0	4	5
		Within-group comparison (%) ^1^	1.9	0.0	7.3	3.1
		Between-group comparison (%) ^2^	20.0	0.0	80.0	100.0
	Not detection	Head count	0	1	0	1
		Within-group comparison (%) ^1^	0.0	1.9	0.0	0.6
		Between-group comparison (%) ^2^	0.0	100.0	0.0	100.0
Bitter threshold	2 mg/L quinine sulphate	Head count	35	24	8	67
		Within-group comparison (%) ^1^	64.8	45.3	14.5	41.4
		Between-group comparison (%) ^2^	52.2	35.8	11.9	100.0
	5 mg/L quinine sulphate	Head count	14	14	24	52
		Within-group comparison (%) ^1^	25.9	26.4	43.6	32.1
		Between-group comparison (%) ^2^	26.9	26.9	46.2	100.0
	10 mg/L quinine sulphate	Head count	5	11	16	32
		Within-group comparison (%) ^1^	9.3	20.8	29.1	19.8
		Between-group comparison (%) ^2^	15.6	34.4	50.0	100.0
	Not detection	Head count	0	4	7	11
		Within-group comparison (%) ^1^	0.0	7.5	12.7	6.8
		Between-group comparison (%) ^2^	0.0	36.4	63.6	100.0
Acid threshold	0.1 g/L tartaric acid	Head count	11	19	13	43
		Within-group comparison (%) ^1^	20.4	35.8	23.6	26.5
		Between-group comparison (%) ^2^	25.6	44.2	30.2	100.0
	0.5 g/L tartaric acid	Head count	26	26	30	82
		Within-group comparison (%) ^1^	48.1	49.1	54.5	50.6
		Between-group comparison (%) ^2^	31.7	31.7	36.6	100.0
	0.75 g/L tartaric acid	Head count	14	8	10	32
		Within-group comparison (%) ^1^	25.9	15.1	18.2	19.8
		Between-group comparison (%) ^2^	43.8	25.0	31.2	100.0
	1 g/L tartaric acid	Head count	2	0	0	2
		Within-group comparison (%) ^1^	3.7	0.0	0.0	1.2
		Between-group comparison (%) ^2^	100.0	0.0	0.0	100.0
	Not detection	Head count	1	0	2	3
		Within-group comparison (%) ^1^	1.9	0.0	3.6	1.9
		Between-group comparison (%) ^2^	33.3	0.0	66.7	100.0

^1^ % in the group with the pertinent threshold at this concentration. ^2^ % of each group with the pertinent threshold at this concentration. ^3^ All subjects (*n* = 162) detected the highest concentration of sweet (20 g/L), but none indicated it as their lower threshold (-), since they indicated one of the other three lower concentrations as their Absolute threshold (AT).

**Table 3:** Astringency detection results by groups.

		Student Group			
Astringency Detection		Group 1	Group 2	Group 3	Total
No detection	Head count	7	0	9	16
	% of student group	13.0	0.0	16.4	9.9
	% of astringency detection	43.8	0.0	56.2	100.0
Astringency detection	Head count	42	11	45	98
	% of student group	77.8	20.8	81.8	60.5
	% of astringency detection	42.9	11.2	45.9	100.0
Named bitter	Head count	2	42	0	44
	% of student group	3.7	79.2	0.0	27.2
	% of astringency detection	4.5	95.5	0.0	100.0
Named acid	Head count	3	0	1	4
	% of student group	5.6	0.0	1.8	2.5
	% of astringency detection	75.0	0.0	25.0	100.0

## Data Availability

The data presented in this study are available on request from the corresponding author.
